# Lymphatic Mapping in the Treatment of Chronic Seroma: A Case Series

**Published:** 2015-02-27

**Authors:** Michael Singer, Kristen Aliano, Steven Stavrides, Thomas Davenport

**Affiliations:** Long Island Plastic Surgical Group, Garden City, NY

**Keywords:** seroma, lymphatic mapping, lymphocele, allergy, arthroplasty

## Abstract

**Objective:** *Seromas* or lymphoceles are common postoperative complications. This series presents 3 patients with lower extremity seromas refractory to treatment that required lymphatic mapping and lymphatic ligation for closure, and in 1 case, diagnosis. **Methods:** Lymphatic mapping procedure consisted of intraoperative injection of subcutaneous tissue with methylene blue distal to the seroma with observation of dye effluence from transected or injured lymphatics draining into area of seroma. **Results:** *In* 2 patients, methylene blue dye absorption into lymphatic vessels allowed for optimized visual identification of lymphatic leak and contrast against surrounding tissues. In the third patient, where no lymphocele leak was found, the study was diagnostic and helped to find an alternate etiology for the recurrent seroma. **Conclusion:** Lymphatic mapping with methylene blue dye is an effective tool in the evaluation and diagnosis of chronic seroma.

Seromas are a mass or tumefaction caused by localized accumulation of serum within tissues or organs that occur secondary to surgery or blunt trauma where significant tracts of lymphatic tissue have been removed, injured, or occluded. This condition is characterized by palpable, fluctuant mass and confirmed by ultrasonography. Serous seepage or impaired lymphatic drainage may result in passive leak into adjacent compartments which may persist. Accumulated serous fluid may require multiple aspirations or drain placement and sometimes surgery to repair the area. Complications include infection, wound dehiscence, or in rare cases calcification of the seroma capsule.^1^ Seromas resolve slowly and are often refractory to conventional treatment with prolonged drainage, multiple aspirations, and local pressure.^2^ Large seromas may cause edema of the extremities, pain, unsatisfactory cosmetic outcomes, and can become a chronic draining wound.^3^^,^^4^

## METHODS

Three patients were treated for symptomatic seroma or lymphocele following surgery. All 3 patients had a longstanding seroma refractory to treatment with dressings, negative pressure vacuum-assisted closure therapy, or surgery to excise and close the seroma capsule. The lymphatic mapping procedure consisted of intraoperative injection of subcutaneous tissue with methylene blue distal to the seroma with observation of dye effluence from transected or injured lymphatics draining into the seroma. Diagnoses were made on the basis of medical history, physical examination, and positive lymphatic mapping findings at the time of operation. A summary of subjects’ demographics, diagnoses, treatment methods, and outcomes can be found in Table I.

## FIRST CASE

**Figure 1:**
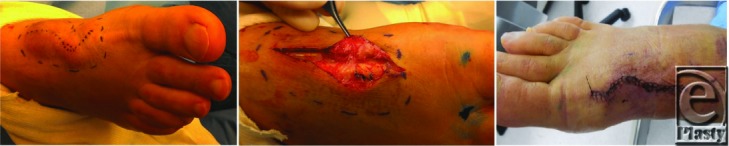
First Patient. A. Pre-operative tracing of lymphatic. B. Intra-operative visualization of dye-injected lymphatic. C. Post-surgical closure and resolution.

The first patient (Fig 1) was a 63-year-old male with a history of dorsal cyst excision of the right foot and subsequent chronic seroma requiring multiple aspirations. The patient had undergone a previous seroma excision and closure with subsequent recurrence. The patient then underwent an excision procedure including lymphatic mapping and ligation of leaking lymphatics. First, the subcutaneous space distal to the seroma was injected with 50% methylene blue dye. Draining lymphatics were identified by their bluish appearance and location of the leak was identified by discharge of dye into the wound. The leaking lymphatic vessel was visualized and ligated with absorbable suture, and no further leaking was observed. No intraoperative complications occurred and no hypersensitivity to methylene blue was noted. A Jackson-Pratt drain was placed to provide aspiration after closure and the patient was taken to the post-anesthesia care unit in stable condition. The drain was removed on post-operative day 3 and there was no recurrence of seroma.

## SECOND CASE

**Figure 2:**
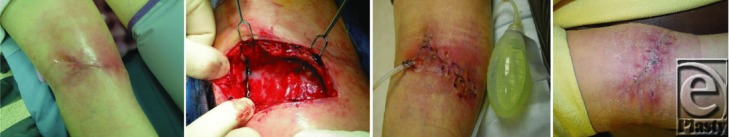
Second patient. A. Pre-operative effluence from incision-site. B. Intra-operative visualization of dye-injected lymphatic. C. Drain placement and closure. D. Post-operative resolution.

The second patient (Fig 2) was an otherwise healthy 70-year-old woman with previous history of biopsy-proven malignant melanoma of the left posterior popliteal fossa. An open draining seroma refractory to treatment with negative pressure therapy and dressings developed subsequent to wide-local excision of the melanoma. The patient was taken to the operating room where lymphatic mapping proceeded with 5 cc methylene blue dye injection into the first web space of the foot. Draining lymphatic vessels were clearly identified by their bluish appearance and were readily preserved. The lymphatic leak was ligated, the wound was closed, and a drain was placed. No intraoperative complications occurred, no hypersensitivity to methylene blue was observed, and the patient was taken to the post-anesthesia care unit in stable condition. The drain was removed on post-operative day 4 and the wound healed well without incident.

## THIRD CASE

**Figure 3:**
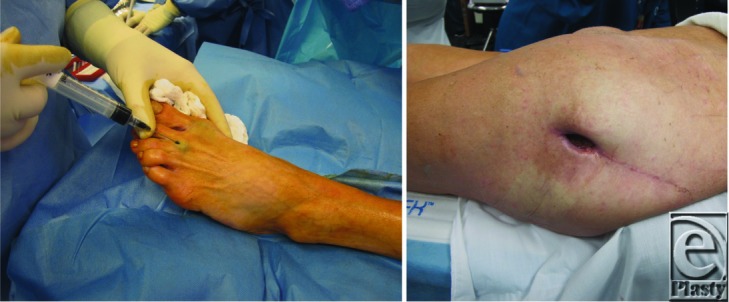
Third patient. A. Intra-operative dye injection of superficial dorsal lymphatic. B. Serous fluid accumulation due to chromium allergy.

The third patient (Fig 3) was a 54-year-old woman presenting with chronic mixed seroma-hematoma post-total hip arthroplasty that persisted after multiple aspirations and 2 excision procedures with drain placement. The patient was taken to the operating room where lymphatic mapping commenced with subcutaneous methylene blue injection into the dorsum of the patient's left foot. Exploration of the wound proceeded through the tensor fascia lata (TFL) and despite the presence of some lymphatics in the wound no lymphatic leak was identified. A deep draining area was found extending to the area beneath the TFL and surrounding the implant. A Jackson-Pratt drain was placed into this area and the TFL was reclosed over the drain. Subsequently, the superficial compartment was excised using a 15 scalpel blade and Bovie cautery and the entire seroma was closed using quilting sutures. Cultures and lymphatic mapping results were negative, indicating a potentially disparate etiology for this chronic seroma that led to refinement of a differential diagnosis. Suspicion of an inflammatory reaction was confirmed by a positive finding after completion of a chromium allergy test. The implanted prosthesis was then exchanged and resolution of the seroma followed.

## RESULTS

Lymphatic mapping in 2 of 3 patients contributed to diagnosis and allowed for enhanced visualization, ligation, and resolution of chronic seromas. In the third patient, lymphatic mapping produced a negative result, thus aiding in refinement of the differential diagnosis. Further assessment of the third patient determined that the chronic seroma was in fact accumulated serous fluid secondary to arthroplasty-derived metal allergy. In all 3 patients, lymphatic mapping led to a diagnosis of either lymphatic leakage or the ruling out of lymphatic leakage, which ultimately led to correct diagnosis of a metal allergy in one patient.

## DISCUSSION

Seroma is a post-surgical complication characterized by accumulation of exudative fluid secondary to undermining of the dermis, dead space formation, shearing force between underlying tissues and flaps, and transection of lymphatic or blood vessels responsible for interstitial fluid absorption.^5^ Incidence of seroma, lymphocele, and lymphorrhea is reduced when preference is given to vertical over oblique incisions, use of quilting sutures, and drain placement.^6^ Preventative techniques include reducing electrocautery usage,^7^ quilting and progressive tension (Baroudi) sutures,^8^^-^10 ligating perforating vessels,^11^ intraoperative fibrin or adhesive use,^12^ corticosteroids,^13^ diuretics,^14^ compression garments, endoscopic harvest, and drainage placement.^9^^,^^15^ However, conflicting evidence exists for the efficacy of prophylactic fibrin sealant,^16^^-^18 quilting sutures,^19^ steroid use,^20^ and sclerotherapy.^21^^-^23 Drain placement may reduce post-operative hematoma but not seroma incidence.^9^^,^^24^ Additional risk factors exist which cannot be controlled by the surgeon, including prior scarring, body mass index, significant weight loss,^25^ voluminous post-surgical drainage,^26^ and excised skin weight.^27^^,^^28^ Complications include infection, wound dehiscence, flap necrosis, and scarring.^29^^,^^30^ Chronic seroma may lead to formation of a fibrous capsule, tissue calcifications, or pseudobursa that is associated with adverse outcomes and often requires surgical intervention.^25^^,^^31^ Therapeutic management of seroma includes initial treatment with serial aspiration and followed by drain placement.^18^ Further options for rectifying seroma include fibrin sealants,^32^^-^34 sclerotherapy,^22^^,^^26^^,^^35^^,^^36^ serial aspiration,^37^^,^^38^ corticosteroid injection,^39^^,^^40^ lymphatic mapping with ligation,^41^^-^43 and surgical capsulectomy.^44^^,^^45^Lymphatic mapping is traditionally used in sentinel-node biopsy when assessing mammary carcinoma progression in patients exhibiting clinically negative axillary lymph nodes while undergoing breast conservation treatment.^46^^,^^47^ Methylene blue is a dye used in histology or as a contrast agent^48^ and is used here similarly to isosulfan blue in intraoperative lymphatic mapping for direct visualization of vessels to assist ligation.^41^^-^43^,^^49^^,^^50^ Lymphatic mapping has also been used to visualize lymphatics for lymph node transfer and lymphovenous anastomosis in the treatment of lymphedema.^51^

In this series, lymphatic mapping allowed for prime visualization of lymphatic leak into seromas. In the third case, lymphatic mapping produced a negative result for seroma, which assisted in refining the differential diagnoses. Further examination determined that seroma was actually serous fluid accumulation from inflammation. Hallab et al^52^ and Thomas et al^53^ reported an increased rate of metal allergy incidence in patients with failed implants.^52^^,^^53^ Though detrimental outcomes are rare, nickel, chromium, and cobalt are known allergens implicated in allergic contact dermatitis, irritant skin-toxicity, delayed hypersensitivity eczema, and lymphocyte-dominant peri-prosthetic inflammation.^53^^-^57

## CONCLUSION

Lymphatic mapping using methylene blue dye is an effective tool in intraoperative visualization of lymphatic vessels and ligation of lymphatic leaks. Prosthesis-derived metal allergy may mimic a chronic lymphatic leak as both can cause edema of the extremities, localized swelling, tenderness, and pain as reported in the literature. While the number of patients in this series is limited, these encouraging results indicate that lymphatic mapping may be useful in both the treatment of chronic seromas and the diagnosis of recurrent fluid collections with confounding etiology.

## Figures and Tables

**Table 1:** Patient demographics, findings, and outcomes

	Patient 1	Patient 2	Patient 3
Age, y	63	64	54
Gender (M/F)	M	F	F
Diagnosis	Recurrent seroma postcyst excision	Seroma postmelanoma excision	Seroma hematoma postarthroplasty
Lymphatic mapping finding (+/−)	+	+	−
Treatment	Ligation	Ligation	N/A
Resolution	No recurrence	No recurrence	Total hip arthroplasty replacement
